# Association Between Chronotype and Cardiometabolic Risk in 1462 Adults from the General Population: Mediation Analysis of Body Fat Percentage and Waist-to-Height Ratio

**DOI:** 10.3390/metabo16040243

**Published:** 2026-04-04

**Authors:** Alexander Javier Iman Torres, Jessy Patricia Vásquez Chumbe, Jorge Armando Sifuentes Da Silva, Roger Ruiz-Paredes, Alenguer Gerónimo Alva Arévalo, Wilson Guerra Sangama, Antonio Castillo-Paredes, Jose Jairo Narrea Vargas

**Affiliations:** 1 Departamento de Ciencia y Tecnología de Alimentos, Facultad de Industrias Alimentarias, Universidad Nacional de la Amazonía Peruana, Iquitos 16001, Peru; alexander.iman@unapiquitos.edu.pe (A.J.I.T.); jessy.vasquez@unapiquitos.edu.pe (J.P.V.C.); sifuentesjorge1990@gmail.com (J.A.S.D.S.); 2 Departamento de Ingeniería de Alimentos, Facultad de Industrias Alimentarias, Universidad Nacional de la Amazonía Peruana, Iquitos 16001, Peru; roger.ruiz@unapiquitos.edu.pe (R.R.-P.); alenguer.alva@unapiquitos.edu.pe (A.G.A.A.); wilson.guerra@unapiquitos.edu.pe (W.G.S.); 3 Grupo AFySE, Investigación en Actividad Física y Salud Escolar, Escuela de Pedagogía en Educación Física, Facultad de Educación, Universidad de Las Américas, Santiago 8370040, Chile; acastillop85@gmail.com; 4 Grupo de Investigación en Nutrición, Metabolismo y Ejercicio, Facultad de Ciencias de la Salud, Carrera de Nutrición y Dietética, Universidad Científica del Sur, Lima 15067, Peru

**Keywords:** circadian rhythm, adiposity, metabolic syndrome, anthropometry

## Abstract

**Introduction:** Circadian misalignment has been proposed as a potential determinant of cardiometabolic risk. Chronotype, as an expression of individual circadian organization, has been associated with unfavorable metabolic profiles; however, the role of total and central adiposity as potential mediating mechanisms in this relationship remains incompletely understood. **Objective:** This study aimed to analyze the association between chronotype and cardiometabolic risk in adults and to evaluate the potential mediating role of body fat percentage (BF%) and waist-to-height ratio (WHtR). **Methods:** An observational study was conducted in 1462 adults from the general population. Chronotype was assessed using the Morningness–Eveningness Questionnaire (MEQ), and cardiometabolic risk was evaluated using a continuous cardiometabolic risk score (CMRS) derived from waist circumference (WC), systolic blood pressure (SBP), triglycerides (TG), fasting blood glucose (FBG), and total cholesterol (TC). Multiple linear regression models adjusted for covariates were used to examine the association between chronotype and CMRS, and hierarchical regression was performed to estimate the incremental contribution of adiposity indicators. Mediation analysis was conducted using the PROCESS macro (Model 4) with 95% bootstrap confidence intervals. **Results:** Chronotype was independently associated with CMRS after adjustment for covariates (β = 0.055; *p* = 0.030), although the effect size and explained variance were small. In hierarchical regression analysis, the inclusion of chronotype explained a small but significant increase in CMRS variance (ΔR^2^ = 0.003; *p* = 0.030). The addition of adiposity indicators significantly increased the explained variance (ΔR^2^ = 0.014; *p* < 0.001), with WHtR emerging as the most relevant predictor in the final model. Bootstrap mediation analysis did not reveal significant indirect effects of BF% or WHtR on the relationship between chronotype and CMRS. In sensitivity analyses excluding waist circumference from the CMRS, the association between chronotype and cardiometabolic risk was no longer significant (β = −0.001; *p* = 0.974). **Conclusions:** Chronotype showed a modest association with cardiometabolic risk in the primary analysis. However, sensitivity analyses indicated that this association may partly depend on the inclusion of waist circumference within the composite cardiometabolic risk score. These findings highlight the central role of abdominal adiposity in cardiometabolic health and suggest that the relationship between chronotype and cardiometabolic risk should be interpreted with caution.

## 1. Introduction

Cardiometabolic diseases represent a major global public health concern, with increasing prevalence across the adult population [1]. Their development results from a complex interaction among metabolic, behavioral, and environmental factors, within which circadian regulation has emerged as a key biological determinant of energy and cardiovascular metabolism [2,3]. Accumulating evidence suggests that misalignment between endogenous circadian rhythms and daily behavioral patterns—including activity, sleep, and eating schedules—is associated with disturbances in glucose–lipid homeostasis, blood pressure regulation, and body composition, thereby contributing to increased cardiometabolic risk [3,4,5,6].

Chronotype, defined as the individual preference for engaging in activities at earlier or later times of the day, represents a relatively stable expression of circadian organization and reflects interindividual differences in the synchronization between endogenous biological clocks and the social environment. Several observational studies have documented that evening chronotype is associated with a less favorable cardiometabolic profile, even in apparently healthy young populations, characterized by greater adiposity, dyslipidemia, and early metabolic alterations [7,8,9]. Among young adults in Europe and the Americas, evening chronotype has been associated with higher triglyceride (TG), total cholesterol (TC), and LDL-cholesterol concentrations, as well as a greater risk of metabolic syndrome, independent of dietary intake [8]. Consistently, prospective studies in Asian populations have confirmed that late chronotype is associated with a longitudinal increase in overall cardiometabolic risk, reinforcing the temporal directionality of this relationship [3].

Total adiposity and, particularly, central fat distribution play a key role in cardiometabolic pathophysiology, given their close association with insulin resistance, dyslipidemia, and hemodynamic alterations [5,10]. In this context, chronotype has been identified as an independent factor associated with abdominal obesity and visceral fat accumulation. In adults, evening chronotype has been linked to higher waist circumference (WC) values and greater abdominal adiposity, even after adjustment for dietary adherence and other lifestyle factors, suggesting a specific circadian effect on fat distribution [11]. Complementarily, recent studies have shown that chronotype is associated with distinct anthropometric and biochemical profiles relevant to cardiovascular risk assessment in individuals with obesity, including variations in lipid and glycemic indicators [9].

Circadian misalignment associated with social and occupational factors, such as night work and rotating shift schedules, has been related to greater global and central adiposity across diverse populations. Studies based on national surveys and occupational cohorts have shown that exposure to night work is associated with a higher likelihood of obesity, elevated WC, and visceral fat accumulation, as well as with the presence of subclinical markers of atherosclerosis [12,13]. Furthermore, the combination of night work and evening chronotype has been linked to increased cardiometabolic risk in specific groups, such as female healthcare workers [14].

Beyond adiposity, recent evidence highlights that disruption of circadian organization, assessed through rest–activity patterns, is associated with higher cardiometabolic risk. In young adults and representative population samples, less stable circadian rhythms have been linked to less favorable metabolic profiles and a higher prevalence of obesity, central adiposity, and hypertension, reinforcing the role of circadian misalignment as a relevant component of cardiometabolic health [2,5].

From a behavioral perspective, evening chronotype has also been associated with misaligned eating patterns, characterized by greater energy intake later in the day, which in turn has been related to an increased risk of obesity and metabolic syndrome [15,16]. In this regard, studies in the field of chrononutrition suggest that the metabolic efficacy of interventions based on the temporal organization of food intake, such as time-restricted eating, may depend on individual circadian preference, which in turn may modulate energy balance, body composition, and metabolic health [17]. Taken together, this evidence suggests that total adiposity and its distribution may act as key intermediate mechanisms in the relationship between chronotype and cardiometabolic risk.

Therefore, the aim of the present study was to analyze the association between chronotype and cardiometabolic risk in a large sample of adults and to explore whether body fat percentage (BF%) and fat distribution statistically account for part of this relationship.

## 2. Materials and Methods

### 2.1. Study Design

A cross-sectional correlational observational study was conducted.

### 2.2. Participants

Participants were adults aged 18–59 years who were recruited from the general population of the districts of Iquitos, Belén, Punchana, and San Juan Bautista in the province of Maynas, department of Loreto, Peru. The target population comprised 235,812 adults according to municipal records for the year 2025 (Iquitos: 90,868; Belén: 35,304; Punchana: 40,997; San Juan Bautista: 68,643).

### 2.3. Sample Size and Eligibility Criteria

The sample was calculated using stratified probabilistic sampling by district, based on the corresponding population size and the formula for finite populations. An expected proportion of 50%, a 95% confidence level (Z = 1.96), and a margin of error of 5% were assumed. Based on these parameters, the estimated sample included 367 adults from Iquitos, 364 from Belén, 365 from Punchana, and 366 from San Juan Bautista, yielding a total sample size of 1462 adults. Sample size calculation was performed using Epi Info software, version 7.2 (Centers for Disease Control and Prevention, Atlanta, GA, USA).

Participant selection within each district was conducted through multistage cluster sampling. Primary sampling units were territorial areas (streets and settlements), and secondary units were households, which were randomly selected.

Inclusion criteria were adults aged 18–59 years, of both sexes, holding a national identity document confirming residence in the selected districts, and willing to participate by signing informed consent. Individuals with physical and/or mental conditions that could compromise data collection were excluded.

### 2.4. Study Variables and Procedures

Participant recruitment involved home visits within previously selected clusters. In each cluster, one to two households were randomly selected using a systematic procedure, depending on accessibility and availability.

In each household, eligibility criteria were verified. Eligible participants received an explanation of the study objectives and signed informed consent prior to data collection. Data collection was conducted exclusively after consent had been obtained.

Data were collected at participants’ homes, with an average duration of approximately 60 min per participant, between April and September 2024.

#### 2.4.1. Anthropometry and Sociodemographic Variables

Anthropometric assessments were performed by trained and standardized researchers under the supervision of a certified anthropometry specialist, following current Peruvian technical guidelines [18].

Body weight was measured with participants barefoot and wearing light clothing using a digital scale (HBF-514C, Omron Healthcare Co., Ltd., Kyoto, Japan), with ±1% precision and automatic calibration. Height was measured using a stadiometer certified by the National Center for Food, Nutrition, and Healthy Living (CENAN, Lima, Peru), with 0.1 cm precision. Body mass index (BMI) was calculated as weight (kg) divided by height squared (m^2^).

Age, sex, smoking status, family history of cardiometabolic disease (FH-CMD), and physical activity level (PAL) were obtained through structured face-to-face interviews conducted at participants’ homes under conditions ensuring privacy and confidentiality.

Age was recorded as a continuous variable. Sex was categorized dichotomously. Smoking status was classified into four categories: current smoker (use within the last month), former smoker (no use in the last month but use within the previous 12 months), passive smoker (no active smoking within the past 12 months but exposure to secondhand smoke), and non-smoker (no active smoking or passive exposure).

FH-CMD was classified as premature (father/brother <55 years and/or mother/sister <65 years with cardiometabolic disease), non-premature (father/brother ≥55 years and/or mother/sister ≥65 years), or no first-degree family history.

PAL was categorized as low, moderate, or high according to reported frequency, duration, and intensity over the previous month. Low PAL was defined as no moderate-intensity activity performed more than 3–4 times per week; moderate PAL as moderate-intensity activity performed 3–4 times per week with a duration <150 min per week; and high PAL as moderate-intensity activity performed ≥30 min per day.

#### 2.4.2. Chronotype

The primary exposure variable was chronotype, assessed using the Morningness–Eveningness Questionnaire (MEQ) developed by Horne and Östberg, a validated 19-item instrument evaluating individual circadian preference regarding sleep, wakefulness, and daily performance [19]. The MEQ has demonstrated adequate internal consistency (Cronbach’s α > 0.80) [20], temporal stability, and construct and convergent validity in diverse populations, including middle-aged adults [21].

Participants were classified for descriptive analyses as morning type (59–86 points), intermediate (42–58 points), or evening type (16–41 points). The full questionnaire is provided as Supplementary Material File S1. Higher MEQ scores indicate a stronger morning preference (greater morningness), whereas lower scores reflect a greater evening preference.

For inferential analyses, chronotype was treated as a continuous variable using the total MEQ score.

#### 2.4.3. Cardiometabolic Risk

The outcome variable was cardiometabolic risk, assessed quantitatively using a continuous cardiometabolic risk score (CMRS). The CMRS was constructed from WC, systolic blood pressure (SBP), TG, fasting blood glucose (FBG), and TC, based on criteria established by international organizations including the World Health Organization, the American Heart Association, the International Diabetes Federation, and the American Diabetes Association [22,23].

The CMRS was calculated as the unweighted sum of the five components included in the score: WC (cm), SBP (mmHg), TG (mg/dL), FBG (mg/dL), and TC (mg/dL). Because all CMRS components were directly proportional to cardiometabolic risk, the composite score was constructed using their raw values without inversion or weighting. No standardization, rescaling, or transformation was applied to these variables before summation. Therefore, the reported CMRS values represent the raw composite score derived from the direct addition of the five components, with higher values indicating greater global cardiometabolic risk.

WC was measured using a metallic measuring tape (Luftkin, Apex Tool Group, LLC, Sparks, MD, USA) at the midpoint between the lower margin of the last palpable rib and the top of the iliac crest [24].

SBP was measured using a digital monitor (HEM-7120-LA, Omron Healthcare Co., Ltd., Kyoto, Japan) after a resting period, ensuring correct cuff placement and arm positioning at heart level [25].

FBG was measured using a portable analyzer (Accu-Chek Instant, Roche Diabetes Care GmbH, Mannheim, Germany). TG and TC were assessed using a portable analyzer (Mission Cholesterol Monitoring System, ACON Laboratories, Inc., San Diego, CA, USA). The procedure included finger antisepsis, sterile lancet puncture, and capillary blood collection [26,27].

BMI was not included as a covariate in the main models to avoid collinearity and overadjustment, given that the CMRS already incorporates an anthropometric measure (WC), and adiposity was explicitly evaluated as a mediator through BF% and waist-to-height ratio (WHtR) [28].

#### 2.4.4. Body Fat Percentage and Waist-to-Height Ratio

The mediating variables were BF% and WHtR, both treated as continuous quantitative variables.

BF% was assessed by bioelectrical impedance analysis using the digital scale (HBF-514C, Omron Healthcare Co., Ltd., Kyoto, Japan), following a standardized protocol including relative fasting, prior bladder emptying, abstinence from vigorous exercise for at least 12 h, and avoidance of caffeine or alcohol within 24 h. Assessments were conducted at similar times of day under controlled conditions [29].

The WHtR was calculated as WC (cm) divided by height (cm). Values >0.50 were considered indicative of elevated central adiposity [30].

Both mediators were treated as continuous variables in inferential analyses.

### 2.5. Ethical Considerations

The study was approved by the Ethics Committee of the Universidad Nacional de la Amazonía Peruana (code: N°018-2024-CIEI-VRINV-UNAP) in accordance with the Declaration of Helsinki [31]. Data confidentiality was ensured in compliance with Peruvian Law N°29733 (Personal Data Protection Law).

### 2.6. Statistical Analysis

Statistical analyses were performed using IBM SPSS Statistics version 22 (IBM Corp., Armonk, NY, USA). All tests were two-tailed, with statistical significance set at *p* < 0.05.

Continuous variables are described using measures of central tendency and dispersion, while categorical variables are expressed as absolute frequencies and percentages. Normality was assessed using the Shapiro–Wilk test and visual inspection of histograms and Q–Q plots.

Descriptive analyses were stratified by sex due to known physiological and metabolic differences. However, multivariable inferential analyses (regression and mediation) were conducted using the total sample, adjusting for sex.

Because continuous variables were not normally distributed, comparisons between two groups (men vs. women) were conducted using the Mann–Whitney U test. Differences in categorical variables, including those with more than two categories (e.g., chronotype classification, smoking status, and physical activity level), were evaluated using Pearson’s chi-square test.

For multivariable analyses, linear regression models were used, given the large sample size (*n* = 1462), which ensures robustness against moderate deviations from normality, particularly when residuals are approximately symmetrically distributed.

Bivariate associations between chronotype score, CMRS, and potential mediators were initially explored using simple linear regression.

Multiple linear regression models were subsequently constructed to evaluate the association between chronotype score and CMRS, adjusted for age, sex, smoking status, FH-CMD, and PAL. Categorical variables (sex, smoking status, FH-CMD, and PAL) were entered into the models using dummy coding, with reference categories defined a priori. Hierarchical regression models were built to examine the incremental contribution of mediators. Model 1 included chronotype score and covariates; Model 2 additionally included BF%; and Model 3 further incorporated WHtR. Results are expressed as unstandardized coefficients (B), standardized coefficients (β), 95% confidence intervals, and *p*-values. Multicollinearity was assessed using variance inflation factors (VIFs), tolerance statistics, condition indices, and pairwise correlations among predictors.

Mediation analysis was conducted using parallel multiple mediation with the PROCESS macro for SPSS (version 4.2), applying Model 4 proposed by Hayes. Indirect effects were estimated using a bootstrap procedure with 5000 resamples. Mediation was considered significant when the 95% confidence interval did not include zero. Models were adjusted for the same covariates used in regression analyses.

**Figure 1:**
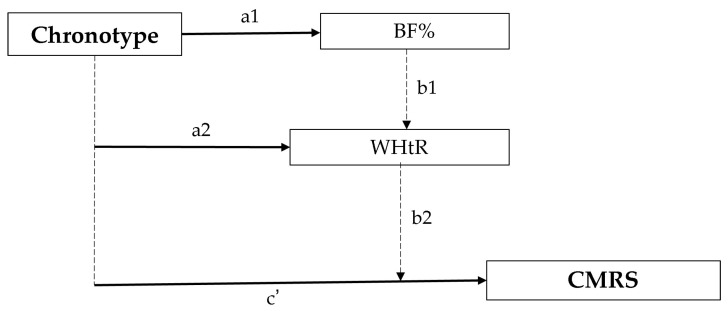
Conceptual mediation model evaluated. Here, a_1_ and a_2_ represent the associations between chronotype and the mediators; b_1_ and b_2_ represent the associations between the mediators and CMRS adjusted for chronotype; and c’ represents the direct association between chronotype and CMRS.

As sensitivity analyses, two additional approaches were performed. First, the association between chronotype and CMRS was examined among participants in the lower and upper quartiles of the MEQ score distribution to evaluate whether the relationship was stronger at the extremes of chronotype. Second, the cardiometabolic risk score was recalculated excluding WC in order to assess whether the association between chronotype and cardiometabolic risk was influenced by the overlapping anthropometric component between the outcome and the mediator (WHtR) (Figure 1).

Finally, a scatter plot with a fitted regression line was generated to visualize the relationship between chronotype score and CMRS in the total sample.

## 3. Results

The sample comprised 1462 participants, of whom 54.2% were men and 45.8% were women. The median age was 38 years (IQR: 28 to 49). Sociodemographic, anthropometric, and lifestyle characteristics of the participants according to sex are presented in Table 1.

Regarding anthropometric variables, the median body weight was 66 kg (IQR: 60 to 72), median height was 1.63 m (IQR: 1.59 to 1.68), and median BMI was 24.5 kg/m^2^ (IQR: 23.3–26.7). Men presented higher values of body weight, height, and WC, as well as a higher WHtR compared with women (*p* < 0.001).

The median chronotype score was 63 points (IQR: 58 to 67). Most participants were classified as morning type (71.8%), followed by intermediate type (27.6%), whereas evening type was infrequent (0.7%). Chronotype distribution differed significantly by sex (*p* = 0.010), with a higher proportion of morning types among men.

Chronotype score was weakly but significantly correlated with age (r = 0.063, *p* = 0.016), indicating a slight tendency toward greater morningness with increasing age (Supplementary Table S1).

Regarding cardiometabolic risk, the median CMRS was 109.1 (IQR: 100.8 to 111.7), with no significant sex differences (*p* = 0.080). The median WC was 87.0 cm (IQR: 80.0 to 90.0), and the median SBP was 110 mmHg (IQR: 100 to 110). Median TG, FBG, and TC levels were 157 mg/dL, 96 mg/dL, and 136 mg/dL, respectively.

Concerning lifestyle factors, 74.0% of participants reported passive exposure to tobacco smoke, 83.5% had a non-premature family history of cardiometabolic disease, and 37.3% reported a high level of physical activity.

In the multiple linear regression model adjusted for age, sex, smoking status, FH-CMD, and PAL (Table 2), with categorical variables entered as dummy variables, chronotype score was significantly associated with CMRS (B = 0.071; β = 0.055; 95% CI: 0.007–0.135; *p* = 0.030). Age emerged as the main independent predictor of CMRS (B = 0.203; β = 0.258; *p* < 0.001). Sex was also significantly associated with CMRS (B = −1.049; β = −0.056; *p* = 0.027), whereas no significant associations were observed for smoking status, FH-CMD, or PAL.

The overall model was statistically significant (F(6,1455) = 19.96; *p* < 0.001) and explained 7.6% of the variance in CMRS (R^2^ = 0.076; adjusted R^2^ = 0.072), indicating a modest explanatory capacity of the model.

Two sensitivity analyses were conducted. First, when the analysis was restricted to participants in the lower and upper quartiles of the MEQ score distribution, the association between chronotype score and CMRS remained statistically significant (β = 0.077, *p* = 0.033), with a slightly larger effect size compared with the main analysis, suggesting that the relationship was not driven by the skewed chronotype distribution in the full sample. Second, when WC was excluded from the CMRS calculation, the association between chronotype and cardiometabolic risk was no longer statistically significant (β = −0.001, *p* = 0.974), indicating that the previously observed association may have been influenced by the shared anthropometric component between the outcome and the mediator.

Hierarchical multiple regression analysis was conducted to evaluate the incremental contribution of chronotype score and adiposity indicators to CMRS (Table 3). Model 1, which included age, sex, smoking status, FH-CMD, and PAL, was statistically significant (R^2^ = 0.073; *p* < 0.001), explaining 7.3% of the variance in CMRS. The inclusion of chronotype in Model 2 significantly increased the explained variance (ΔR^2^ = 0.003; *p* = 0.030). Finally, the addition of BF% and WHtR in Model 3 further improved the model’s explanatory capacity (ΔR^2^ = 0.014; *p* < 0.001), reaching a total R^2^ of 0.090 (adjusted R^2^ = 0.085).

In the final model (Table 4), with categorical variables entered as dummy variables, age (β = 0.257; *p* < 0.001), chronotype score (β = 0.053; *p* = 0.038), and WHtR (β = 0.127; *p* < 0.001) were positively and independently associated with CMRS. BF% was not significantly associated with the outcome (*p* = 0.795), nor were sex, smoking status, FH-CMD, or PAL. No problematic multicollinearity was detected among the predictors included in Model 3. Variance inflation factors ranged from 1.02 to 1.47, and tolerance values ranged from 0.68 to 0.98. Pairwise correlations among predictors were generally low to moderate, with the strongest correlation observed between BF% and WHtR (r = 0.51). Complete collinearity diagnostics are presented in Supplementary Table S2.

A multiple mediation analysis was conducted using PROCESS Model 4 to examine whether BF% and WHtR mediated the association between chronotype score and CMRS (Table 5). The indirect effects through BF% (β = 0.0006; 95% CI: −0.0061 to 0.0059) and WHtR (β = 0.0026; 95% CI: −0.0077 to 0.0128) were not statistically significant, as the confidence intervals included zero. Similarly, the total indirect effect did not reach statistical significance (β = 0.0031; 95% CI: −0.0091 to 0.0139).

**Figure 2:**
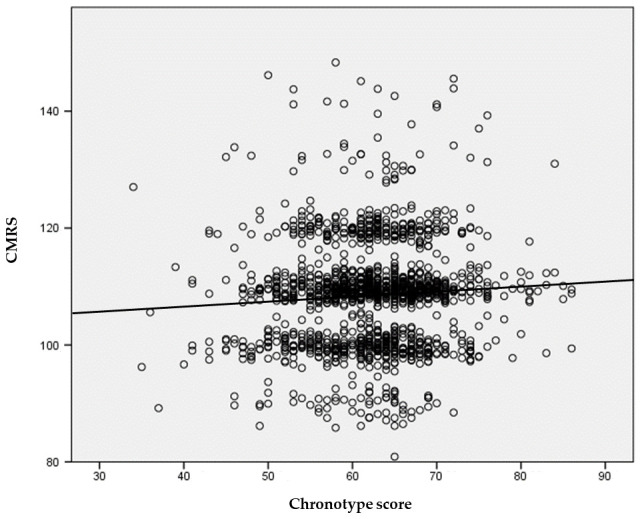
Scatter plot illustrating the association between chronotype score and CMRS in the total sample.

Figure 2 presents the scatter plot depicting the relationship between chronotype score and CMRS. A slight positive linear trend can be observed, consistent with the small standardized coefficient reported in the final model (β = 0.053; *p* = 0.038). However, the substantial dispersion of data points around the regression line suggests a small effect magnitude, which aligns with the minimal increase in explained variance (ΔR^2^ = 0.003) and the reduced effect size observed in the final model.

## 4. Discussion

In this large sample of adults, chronotype showed a modest but statistically significant association with cardiometabolic risk, even after adjustment for age, sex, smoking status, FH-CMD, and PAL. However, the magnitude of the effect was small, and the overall explained variance of the model remained limited. This suggests that although chronotype may contribute to the cardiometabolic profile, its clinical relevance as an isolated predictor is likely modest compared with established metabolic determinants.

The hierarchical analysis showed that the inclusion of chronotype resulted in a statistically significant but modest increase in the explained variance of cardiometabolic risk. Subsequently, the addition of BF% and WHtR substantially improved the explanatory capacity of the model, highlighting the central role of total and central adiposity in determining cardiometabolic risk.

Mediation analyses did not provide evidence that BF% or WHtR statistically explained the association between chronotype and cardiometabolic risk. However, given the cross-sectional design of the study, these analyses should be interpreted as exploratory and do not allow for causal inference.

Our findings are consistent with observational studies documenting an association between chronotype and cardiometabolic profile [3,8,9]. Interestingly, the direction of the association observed in the present study indicates that higher MEQ scores (greater morningness) were associated with slightly higher cardiometabolic risk. At first glance, this finding may appear inconsistent with much of the existing literature, which generally reports a less favorable metabolic profile among individuals with an evening chronotype. However, several factors may help explain this apparent discrepancy. First, the association between chronotype and cardiometabolic health may vary according to population characteristics, including age distribution and sociocultural context. In the present study, age was the strongest predictor of CMRS, and chronotype showed a weak positive correlation with age, suggesting a tendency toward greater morningness in older participants. Because cardiometabolic risk increases with age, part of the observed association between morningness and CMRS may reflect age-related metabolic changes rather than a direct adverse effect of morning chronotype. Second, the distribution of chronotypes in this sample was markedly skewed toward morning types, with very few participants classified as evening types. In such contexts, analyses based on continuous MEQ scores may capture subtle differences within the morning–intermediate spectrum rather than true contrasts between extreme chronotypes, which may partially influence the direction and magnitude of the observed association.

Similarly, research in adult populations has reported that a late chronotype is associated with greater abdominal adiposity and a poorer lipid profile, even after adjustment for dietary and behavioral factors [11].

However, unlike some studies suggesting that adiposity may partially explain the relationship between chronotype and metabolic alterations [5], no significant mediating effect was observed in our sample. This finding should be interpreted cautiously. Given the relatively small effect size of chronotype and the modest proportion of variance explained by this variable, the statistical power to detect indirect effects may have been limited. Therefore, the absence of statistically significant mediation in this dataset does not necessarily rule out a potential role of adiposity in the relationship between chronotype and cardiometabolic risk. Instead, it may reflect limited power to detect small indirect effects or measurement imprecision in the mediating variables.

An approximate post-hoc power analysis was conducted using G*Power (version 3.1) with the “linear multiple regression: fixed model, R^2^ increase” procedure. Based on the observed incremental effect of chronotype (ΔR^2^ = 0.003; f^2^ = 0.00325), a sample size of 1462, and α = 0.05, the achieved statistical power was 0.59. This indicates that, despite the large sample size, the ability to detect very small effects remains limited. Therefore, the absence of statistically significant indirect effects in the mediation models should be interpreted cautiously.

Additional sensitivity analyses provided further insight into the robustness and interpretation of the observed association. When the analysis was restricted to participants in the lower and upper quartiles of the MEQ score distribution, the association between chronotype and CMRS remained statistically significant and slightly stronger than in the main model, suggesting that the relationship was not driven by the skewed distribution of chronotype in the full sample. However, when WC was excluded from the CMRS calculation, the association was no longer statistically significant. This finding suggests that the association observed in the primary analysis may partly depend on the inclusion of WC within the composite cardiometabolic risk score. Because WC represents a major determinant of cardiometabolic risk and is mathematically related to WHtR, this overlap may have contributed to the observed relationship. Therefore, the association between chronotype and cardiometabolic risk should be interpreted cautiously and may partly reflect the contribution of central adiposity rather than a fully independent circadian effect.

In this regard, recent research has shown that chronotype is associated with differences in vascular insulin sensitivity, pancreatic β-cell function, and metabolic flexibility, particularly in individuals with obesity [32,33,34]. These mechanisms may help explain the direct association observed in our study.

Additionally, the literature on circadian misalignment—including night work and exposure to artificial light at night—has demonstrated independent associations with metabolic syndrome and central obesity [35,36,37], reinforcing the hypothesis that circadian disruption may influence cardiometabolic risk through multiple biological and environmental pathways.

Regarding evidence in the Peruvian context, research remains limited. However, the present findings are consistent with results reported in other Latin American settings. In Brazil, individuals with evening chronotypes have shown a higher BMI and a greater prevalence of cardiovascular diseases compared with morning chronotypes [38]. Complementarily, recent studies in Brazilian adults have indicated that chronotype emerges as a key determinant of meal timing, with greater breakfast omission and later food intake as eveningness increases—behavioral patterns previously linked to less favorable metabolic profiles [39]. Likewise, among Latino populations residing in the United States, a significant association between chronotype and metabolic syndrome has been reported, with age-dependent effects suggesting that the impact of chronotype on cardiometabolic risk may vary across the life course [40]. Collectively, these studies support the external plausibility of our findings and suggest that the association between chronotype and cardiometabolic risk is consistent across Latin American populations, despite sociocultural and contextual differences.

The marked predominance of morning chronotypes observed in our sample deserves consideration. Although the low proportion of evening types limits direct comparisons with studies conducted in highly urbanized or student populations, chronotype distribution may be influenced by environmental and sociobehavioral factors, including daily schedules, lifestyle patterns, and exposure to natural light–dark cycles. Previous studies in adult populations from Latin America have also shown that chronotype is associated with behavioral and lifestyle characteristics that may shape circadian preferences [38].

Chronotype distribution may also be influenced by geographic and environmental factors. Populations living closer to the equator experience relatively stable light–dark cycles throughout the year, with minimal seasonal variation in photoperiod. This environmental stability may promote stronger circadian entrainment and has been associated with an earlier chronotype orientation compared with populations living at higher latitudes, where larger seasonal changes in day length tend to favor later chronotypes. Evidence from large population-based studies across different latitudinal gradients has shown that eveningness becomes more prevalent as latitude increases, suggesting that environmental light exposure plays an important role in shaping circadian preference [41].

### 4.1. Practical Implications

In this context, the findings of the present study provide relevant baseline evidence for the design and personalization of time-restricted eating interventions. Although the magnitude of the association between chronotype and cardiometabolic risk was small, its persistence after adjustment for total and central adiposity suggests that chronotype may reflect interindividual differences in daily metabolic organization that could be relevant when designing or tailoring timing-based dietary interventions.

Recent studies have shown that the metabolic efficacy of time-restricted eating is not uniform and may depend on the alignment between eating schedules and individual circadian preference, particularly with respect to insulin sensitivity, glycemic control, and energy balance [42,43]. In this sense, our results suggest that chronotype may be considered a useful baseline marker for population stratification and for guiding the temporal personalization of nutritional interventions, rather than an isolated determinant of cardiometabolic risk.

However, given the observational design of the study, these findings should be interpreted as hypothesis-generating evidence. They support the need for clinical trials and longitudinal studies to evaluate whether adapting time-restricted eating protocols to individual chronotype improves adherence and cardiometabolic outcomes compared with non-personalized approaches.

### 4.2. Limitations and Strengths

Among the main limitations of this study is its cross-sectional design, which precludes the establishment of causal relationships or inference of temporal directionality between chronotype and cardiometabolic risk. Additionally, chronotype was assessed using a self-report instrument, which may introduce measurement bias. Although analyses were adjusted for several relevant covariates—including age, sex, smoking status, FF-CMD, and PAL—residual confounding from unmeasured factors cannot be ruled out. In particular, sleep-related variables such as sleep duration and sleep quality, as well as dietary patterns or timing of food intake, were not available in the dataset and therefore could not be included in the statistical models. These factors are known to influence both chronotype and cardiometabolic health [44].

In addition, although mediation analysis was conducted to explore potential pathways linking chronotype and cardiometabolic risk, the cross-sectional nature of the data does not allow conclusions about causal mechanisms or temporal ordering among the variables.

Another limitation is the markedly skewed distribution of chronotype in the sample, with a predominance of morning-type participants and a very low proportion of evening types. This imbalance may have reduced the statistical power to detect associations related to extreme eveningness and may have attenuated the indirect effects estimated in the mediation models.

Despite these limitations, the study has several notable strengths. These include the large sample size, the use of a CMRS, and the simultaneous evaluation of both total and central adiposity as potential mediators of the observed association. Furthermore, the hierarchical analytical approach allowed for the quantification of the incremental contribution of chronotype beyond traditionally considered sociodemographic and behavioral factors, thereby strengthening the interpretation of the findings.

Regarding the available evidence in the Peruvian and broader Latin American context, the limited number of prior studies examining the association between chronotype and cardiometabolic risk restricts direct comparison of results. However, this limitation also represents a strength, as the present study provides original evidence from an underexplored Latin American setting within the field of chronobiology, contributing to the geographic generalizability of existing knowledge in this area.

### 4.3. Future Perspectives

Longitudinal studies are needed to determine the temporal directionality of the association between chronotype and cardiometabolic risk. Additionally, research integrating objective circadian biomarkers, actigraphy-based sleep parameters, and dynamic metabolic assessments could help clarify the underlying physiological mechanisms.

Furthermore, it would be relevant to explore potential sex differences and interactions with genetic variants related to the circadian clock, as well as to evaluate the effectiveness of personalized chronobiological interventions in reducing cardiometabolic risk.

## 5. Conclusions

In conclusion, chronotype showed a modest association with cardiometabolic risk in adults in the primary analysis. However, sensitivity analyses indicate that this association may partly depend on the inclusion of WC within the cardiometabolic risk score. These findings highlight the central role of abdominal adiposity in cardiometabolic health and suggest that the relationship between chronotype and cardiometabolic risk should be interpreted with caution.

## Figures and Tables

**Table 1:** Sociodemographic, anthropometric, clinical, and lifestyle characteristics of participants according to sex.

Variable		Total (n = 1462)	Men (n = 792)	Women (n = 670)	*p*-Value
		Median [IQR]/n (%)			
Age	years	38 [28–49]	38 [29–50]	37 [28–48]	0.090
Weight	kg	66 [60–72]	68 [65–77]	61 [58–68]	<0.001
Height	cm	1.63 [1.59–1.68]	1.68 [1.63–1.70]	1.59 [1.58–1.61]	<0.001
BMI	kg/m^2^	24.5 [23.3–26.7]	24.6 [23.4–27.1]	23.1 [22.5–24.2]	<0.001
Chronotype	score	63.0 [58.0–67.0]	62.0 [57.0–66.0]	63.0 [59.0–67.0]	0.002
Chronotype type	Morning	1049 (71.8)	593 (74.9)	456 (68.1)	0.010
	Intermediate	403 (27.6)	193 (24.4)	210 (31.3)	
	Evening	10 (0.7)	6 (0.8)	4 (0.6)	
CMRS	-	109.1 [100.8–111.7]	109.1 [101.3–111.9]	108.9 [100.4–111.5]	0.080
WC	cm	87.0 [80.0–90.0]	90.0 [87.0–92.0]	80.3 [79.0–86.0]	<0.001
SBP	mmHg	110.0 [100.0–110.0]	110.0 [100.0–110.0]	110.0 [100.0–110.0]	0.029
TG	mg/dL	157.0 [155.0–161.0]	157.0 [155.0–161.0]	158.0 [155.0–161.0]	0.176
FBG	mg/dL	96.0 [90.0–99.0]	96.0 [90.0–99.0]	95.0 [90.0–98.0]	<0.001
TC	mg/dL	136.0 [128.0–151.0]	138.0 [129.0–158.0]	135.5 [128.0–148.0]	<0.001
WHtR	-	0.53 [0.50–0.55]	0.54 [0.52–0.55]	0.51 [0.49–0.53]	<0.001
BF	%	30.1 [28.7–31.0]	30.3 [29.3–31.3]	29.9 [28.0–30.9]	<0.001
Smoking Status	Non-smoker	-	-	-	0.070
	Passive smoker	1082 (74.0)	605 (76.4)	477 (71.2)	
	Former smoker	61 (4.2)	30 (3.8)	31 (4.6)	
	Current smoker	319 (21.8)	157 (19.8)	162 (24.2)	
FH-CMD	No history	-	-	-	0.170
	Non-premature history	1221 (83.5)	671 (84.7)	550 (82.1)	
	Premature history	241 (16.5)	121 (15.3)	120 (17.9)	
PAL	Low	383 (26.2)	214 (27.0)	169 (25.2)	0.050
	Moderate	534 (36.5)	305 (38.5)	229 (34.2)	
	High	545 (37.3)	273 (34.5)	272 (40.6)	

*p*-Values correspond to comparisons between two groups (men vs. women), using Pearson’s chi-square test for categorical variables and the Mann–Whitney U test for continuous variables. BMI = body mass index; CMRS = cardiometabolic risk score; WC = waist circumference; SBP = systolic blood pressure; TG = triglycerides; FBG = fasting blood glucose; TC = total cholesterol; WHtR = waist-to-height ratio; BF = body fat; FH-CMD = family history of cardiometabolic disease; PAL = physical activity level.

**Table 2:** Multiple linear regression model of CMRS as a function of chronotype score and covariates in adult participants.

Variable	B	95% CI for B	β	*p*-Value	VIF
Chronotype (score)	0.071	0.007–0.135	0.055	0.030	1.013
Age (years)	0.203	0.164–0.242	0.258	<0.001	1.011
Sex (female vs. male)	−1.049	−1.977–−0.120	−0.056	0.027	1.018
Smoking status (vs. passive smoker)	0.130	−0.466–0.726	0.012	0.669	1.158
FH-CMD (vs. non-premature history)	0.453	−0.217–1.123	0.036	0.185	1.176
PAL (vs. high PAL)	0.183	−0.447–0.813	0.016	0.568	1.175

FH-CMD = family history of cardiometabolic disease; PAL = physical activity level; B = unstandardized coefficient; β = standardized coefficient; 95% CI = 95% confidence interval; VIF = variance inflation factor. Multiple linear regression model with CMRS as the dependent variable. Smoking status, FH-CMD, and PAL were entered as dummy variables; passive smoker, non-premature family history of cardiometabolic disease, and high physical activity level were used as reference categories, respectively. Sex was coded as 0 = male and 1 = female. Model statistics: F(6,1455) = 19.96; R^2^ = 0.076; adjusted R^2^ = 0.072; *p* < 0.001. Statistical significance was set at *p* < 0.05.

**Table 3:** Hierarchical multiple regression analysis for CMRS in adult participants.

Model	Variables Included	R^2^	Adjusted R^2^	ΔR^2^	F (Model)	*p* (Model)	*p* (ΔR^2^)
Model 1	Age, sex, smoking status, FH-CMD, PAL	0.073	0.07	—	22.95	<0.001	—
Model 2	Model 1 + Chronotype	0.076	0.072	0.003	19.96	<0.001	0.03
Model 3	Model 2 + BF% + WHtR	0.09	0.085	0.014	17.96	<0.001	<0.001

FH-CMD = family history of cardiometabolic disease; PAL = physical activity level; BF% = body fat percentage; WHtR = waist-to-height ratio. R^2^ represents the cumulative variance explained by the model. ΔR^2^ represents the incremental increase in explained variance after the inclusion of additional variables. All models were adjusted for age, sex, smoking status, family history of cardiometabolic disease, and physical activity level. Categorical variables were entered using dummy coding.

**Table 4:** Coefficients of the final regression model (Model 3) in adult participants.

Variable	B	β	95% CI	*p*-Value
Chronotype (score)	0.068	0.053	0.004–0.131	0.037
Age (years)	0.202	0.257	0.163–0.241	<0.001
Sex (female vs. male)	−0.435	−0.023	−1.392–0.523	0.373
Smoking status (vs. passive smoker)	0.174	0.016	−0.418–0.767	0.564
PAL (vs. high PAL)	0.112	0.01	−0.515–0.738	0.727
FH-CMD (vs. non-premature history)	0.516	0.041	−0.150–1.182	0.129
BF%	−0.022	−0.008	−0.191–0.146	0.795
WHtR	25.901	0.127	13.747–38.056	<0.001

FH-CMD = family history of cardiometabolic disease; PAL = physical activity level; BF% = body fat percentage; WHtR = waist-to-height ratio; B = unstandardized coefficient; β = standardized coefficient; 95% CI = 95% confidence interval. This model corresponds to Model 3 of the hierarchical regression analysis. Sex was coded as 0 = male and 1 = female. Smoking status, FH-CMD, and PAL were entered as dummy variables; passive smoker, non-premature family history of cardiometabolic disease, and high physical activity level were used as reference categories, respectively.

**Table 5:** Multiple mediation analysis of the effect of chronotype score on CMRS through BF% and WHtR (PROCESS Model 4).

Mediator	Indirect Effect	95% Bootstrap CI
BF%	0.0006	−0.0061–0.0059
WHtR	0.0026	−0.0077–0.0128
Total indirect effect	0.0031	−0.0091–0.0139

Indirect effects were estimated using bootstrap procedures with 5000 resamples. Evidence of mediation was considered when the 95% confidence interval did not include zero. All models were adjusted for age, sex, smoking status, family history of cardiometabolic disease, and physical activity level. Categorical covariates were entered using dummy coding.

## Data Availability

The raw data supporting the conclusions of this article are not publicly available due to ethical and privacy restrictions related to the protection of participants’ personal data; however, they are available from the authors upon reasonable request.

## Supplementary Materials

The following supporting information can be downloaded at: https://www.mdpi.com/article/10.3390/metabo16040243/s1, File S1: Morningness–Eveningness Questionnaire; File S2: Ethical Approval Letter; File S3: Written informed consent; Table S1: Pairwise correlations among predictors; Table S2: Collinearity diagnostics for predictors included in Model 3.

## Abbreviations

The following abbreviations are used in this manuscript:

BMI	Body mass index
CMRS	Cardiometabolic risk score
MEQ	Morningness–Eveningness Questionnaire
FH-CMD	Family history of cardiometabolic disease
PAL	Physical activity level
WC	Waist circumference
SBP	Systolic blood pressure
TG	Triglycerides
FBG	Fasting blood glucose
TC	Total cholesterol
WHtR	Waist-to-height ratio
BF%	Body fat percentage

## References

[1] World Health Organization (2023). Cardiovascular Diseases (CVDs).

[2] Hoopes E.K., Witman M.A., D’Agata M.N., Berube F.R., Brewer B., Malone S.K., Grandner M.A., Patterson F. (2021). Rest–activity rhythms in emerging adults: Implications for cardiometabolic health. Chronobiol. Int..

[3] Li T., Xie Y., Tao S., Zou L., Yang Y., Tao F., Wu X. (2023). Prospective study of the association between chronotype and cardiometabolic risk among Chinese young adults. BMC Public Health.

[4] Wanigasinghe A.S., Perera D.S., Rathnayake K.M. (2025). Elevated cardiometabolic risk markers in evening chronotype shift workers: A case-control study in male workers. Br. J. Nutr..

[5] Makarem N., German C.A., Zhang Z., Diaz K.M., Palta P., Duncan D.T., Castro-Diehl C., Shechter A. (2024). Rest-Activity Rhythms Are Associated with Prevalent Cardiovascular Disease, Hypertension, Obesity, and Central Adiposity in a Nationally Representative Sample of US Adults. J. Am. Heart Assoc..

[6] Shafer B.M., Kogan S.A., Rice S.P.M., Shea S.A., Olson R., McHill A.W. (2025). Circadian alignment, cardiometabolic disease, and sex-specific differences in adults with overweight/obesity. J. Clin. Endocrinol. Metab..

[7] Ilkay H.O. (2025). Evening chronotype, irregular circadian eating patterns, and eating-related behaviors may be associated with increased obesity risk among Turkish university students: A large-scale cross-sectional study. Nutr. Res..

[8] Aguilar-Galarza A., García-Gasca T., Mejía C., Díaz-Muñoz M., Pérez-Mendoza M., Anaya-Loyola M.A., Garaulet M. (2021). Evening chronotype is associated with increased triglyceride levels in young adults in two independent populations. Clin. Nutr..

[9] Rabaça Alexandre M., Poínhos R., Oliveira B.M.P.M., Correia F., CRi-O Group (2025). Chronotype, lifestyles, and anthropometric and biochemical indices for cardiovascular risk assessment among obese individuals. Nutrients.

[10] Tałałaj M., Bogołowska-Stieblich A., Wąsowski M., Sawicka A., Jankowski P. (2023). The influence of body composition and fat distribution on circadian blood pressure rhythm and nocturnal mean arterial pressure dipping in patients with obesity. PLoS ONE.

[11] De Amicis R., Galasso L., Leone A., Vignati L., De Carlo G., Foppiani A., Montaruli A., Roveda E., Cè E., Esposito F. (2020). Is abdominal fat distribution associated with chronotype in adults independently of lifestyle factors?. Nutrients.

[12] Correia F.G.S., Ferreira M.J.M., Giatti L., Camelo L.V., Araújo L.F. (2020). Night work is related to higher global and central adiposity in Brazil: National health survey, 2013. Am. J. Ind. Med..

[13] Sugiura T., Dohi Y., Takagi Y., Yoshikane N., Ito M., Suzuki K., Nagami T., Iwase M., Seo Y., Ohte N. (2020). Impacts of lifestyle behavior and shift work on visceral fat accumulation and the presence of atherosclerosis in middle-aged male workers. Hypertens. Res..

[14] Ritonja J., Tranmer J., Aronson K.J. (2019). The relationship between night work, chronotype, and cardiometabolic risk factors in female hospital employees. Chronobiol. Int..

[15] Jeong S., Lee H., Jung S., Kim J.Y., Park S. (2023). Higher Energy Consumption in the Evening Is Associated with Increased Odds of Obesity and Metabolic Syndrome: Findings from the 2016–2018 Korea National Health and Nutrition Examination Survey (7th KNHANES). Epidemiol. Health.

[16] Dote-Montero M., Acosta F.M., Sanchez-Delgado G., Merchan-Ramirez E., Amaro-Gahete F.J., Labayen I., Ruiz J.R. (2023). Correction to: Association of Meal Timing with Body Composition and Cardiometabolic Risk Factors in Young Adults. Eur. J. Nutr..

[17] Reytor-González C., Simancas-Racines D., Román-Galeano N.M., Annunziata G., Galasso M., Zambrano-Villacres R., Verde L., Muscogiuri G., Frias-Toral E., Barrea L. (2025). Chrononutrition and energy balance: How meal timing and circadian rhythms shape weight regulation and metabolic health. Nutrients.

[18] Ministerio de Salud (Perú) (2012). Guía Técnica Para la Valoración Nutricional Antropométrica de la Persona Adulta.

[19] Horne J.A., Ostberg O. (1976). A self-assessment questionnaire to determine morningness–eveningness in human circadian rhythms. Int. J. Chronobiol..

[20] Di Milia L., Adan A., Natale V., Randler C. (2013). Reviewing the psychometric properties of contemporary circadian typology measures. Chronobiol. Int..

[21] Taillard J., Philip P., Chastang J.F., Bioulac B. (2004). Validation of Horne and Ostberg morningness–eveningness questionnaire in a middle-aged population of French workers. J. Biol. Rhythms.

[22] Arnett D.K., Blumenthal R.S., Albert M.A., Buroker A.B., Goldberger Z.D., Hahn E.J., Himmelfarb C.D., Khera A., Lloyd-Jones D., McEvoy J.W. (2019). 2019 ACC/AHA guideline on the primary prevention of cardiovascular disease: Executive summary: A report of the American College of Cardiology/American Heart Association task force on clinical practice guidelines. Circulation.

[23] Kassi E., Pervanidou P., Kaltsas G., Chrousos G. (2011). Metabolic syndrome: Definitions and controversies. BMC Med..

[24] World Health Organization (2011). Waist Circumference and Waist–Hip Ratio: Report of a WHO Expert Consultation, Geneva, 8–11 December 2008.

[25] World Health Organization (2020). WHO Technical Specifications for Automated Non-Invasive Blood Pressure Measuring Devices with Cuff.

[26] International Diabetes Federation (2012). Global Guideline for Type 2 Diabetes.

[27] Foundation for Innovative New Diagnostics (FIND) (2020). Landscape of Point-Of-Care Diagnostics for Cardiometabolic Diseases.

[28] Ashwell M., Gunn P., Gibson S. (2012). Waist-to-height ratio is a better screening tool than waist circumference and BMI for adult cardiometabolic risk factors: Systematic review and meta-analysis. Obes. Rev..

[29] Kyle U.G., Bosaeus I., De Lorenzo A.D., Deurenberg P., Elia M., Gómez J.M., Heitmann B.L., Kent-Smith L., Melchior J.C., Pirlich M. (2004). Bioelectrical impedance analysis—Part II: Utilization in clinical practice. Clin. Nutr..

[30] Yoo E.G. (2016). Waist-to-height ratio as a screening tool for obesity and cardiometabolic risk. Korean J. Pediatr..

[31] World Medical Association (2013). World Medical Association declaration of Helsinki: Ethical principles for medical research involving human subjects. JAMA.

[32] Malin S.K., Remchak M.E., Heiston E.M., Battillo D.J., Gow A.J., Shah A.M., Liu Z. (2024). Intermediate versus morning chronotype has lower vascular insulin sensitivity in adults with obesity. Diabetes Obes. Metab..

[33] Malin S.K., Remchak M.E., Heiston E.M., Fabris C., Shah A.M. (2025). Pancreatic β-cell function is higher in morning versus intermediate chronotypes with obesity. Obes. Sci. Pract..

[34] Remchak M.E., Heiston E.M., Ballantyne A., Dotson B.L., Stewart N.R., Spaeth A.M., Malin S.K. (2022). Insulin sensitivity and metabolic flexibility parallel plasma TCA levels in early chronotype with metabolic syndrome. J. Clin. Endocrinol. Metab..

[35] Cheng W.J., Liu C.S., Hu K.C., Cheng Y.F., Karhula K., Härmä M. (2021). Night shift work and the risk of metabolic syndrome: Findings from an 8-year hospital cohort. PLoS ONE.

[36] Lin S.C., Yeh W.C., Liu Z.X., Hsu H.F., Chen J.Y. (2025). Night-shift work and its association with metabolic syndrome. Medicine.

[37] Hu L.W., Gong Y.C., Zou H.X., Wang L.B., Sun Y., Godinez A., Yang H.Y., Wu S.H., Zhang S., Huang W.Z. (2024). Outdoor light at night is a modifiable environmental factor for metabolic syndrome: The 33 Communities Chinese Health Study (33CCHS). Sci. Total Environ..

[38] Reis-Canaan J.C., Canaan M.M., Costa P.D., Rodrigues-Juliatte T.P., Pereira M.C.A., Castelo P.M., Pardi V., Murata R.M., Pereira L.J. (2021). Association between Chronotype and Nutritional, Clinical and Sociobehavioral Characteristics of Adults Assisted by a Public Health Care System in Brazil. Nutrients.

[39] Longo-Silva G., Serenini R., Lima M.O., Melo J.S., Soares L.L., Menezes R.C.E. (2025). Chrononutrition: Exploring determinants and feature importance of eating timing among Brazilian adults. Cien. Saude Colet..

[40] Maghsoudipour M., Allison M.A., Patel S.R., Talavera G.A., Daviglus M., Zee P.C., Reid K.J., Makarem N., Malhotra A. (2022). Associations of chronotype and sleep patterns with metabolic syndrome in the Hispanic community health study/study of Latinos. Chronobiol. Int..

[41] Randler C., Rahafar A. (2017). Latitude affects morningness–eveningness: Evidence for the environment hypothesis based on a systematic review. Sci. Rep..

[42] Świątkiewicz I., Nuszkiewicz J., Wróblewska J., Nartowicz M., Sokołowski K., Sutkowy P., Rajewski P., Buczkowski K., Chudzińska M., Manoogian E.N.C. (2024). Feasibility and cardiometabolic effects of time-restricted eating in patients with metabolic syndrome. Nutrients.

[43] Malin S.K., Syeda U.S.A., Remchak M.E., Heiston E.M. (2024). Early chronotype favors appetite and reduced later-day caloric intake among adults with obesity. Chronobiol. Int..

[44] Rosi A., Lotti S., Vitale M., Pagliai G., Madarena M.P., Bonaccio M., Esposito S., Ferraris C., Guglielmetti M., Angelino D. (2022). Association between chronotype, sleep pattern, and eating behaviours in a group of Italian adults. Int. J. Food Sci. Nutr..

