# Supplementary material for: Association Between Chronotype and Cardiometabolic Risk in 1462 Adults from the General Population: Mediation Analysis of Body Fat Percentage and Waist-to-Height Ratio
Source: Metabolites. 2026 Apr 4;16(4):243. doi: 10.3390/metabo16040243 (PMC13118073; doi:10.3390/metabo16040243)
Supplement: Supplementary file 1 [file metabolites-16-00243-s001.zip › Supplementary Material S1. Morningness-Eveningness Questionnaire.pdf]

Horne and Östberg Morningness- Eveningness Questionnaire

IDENTIFIER

Identification code ☐ ☐

Hour ☐ **Sector** ☐ **Area** ☐

PARTICIPANT DATA

Name ☐

Sex ☐ **Age** ☐ **Occupation** ☐

For each question, please select the answer that best describes your situation by marking it with a cross in the corresponding box. Answer based on how you have felt in recent weeks.

1. If you only thought about when you would feel better and were completely free to plan your day, what time would you get up?
   5 ☐ Between 05:00 *(5 AM)* and 06:30 *(6:30 AM)* in the morning
   4 ☐ Between 06:30 *(6:30 AM)* and 07:45 *(7:45 AM)* in the morning
   3 ☐ Between 07:45 *(7:45 AM)* and 09:45 *(9:45 AM)* in the morning
   2 ☐ Between 09:45 *(9:45 AM)* and 11:00 *(11 AM*) in the morning
   1 ☐ Between 11 *AM* and *12* PM (Noon)

2. If you only thought about when you would feel better and were completely free to plan your day, what time would you go to bed?
   5 ☐ At *8 PM – 9 PM*
   4 ☐ At *9 PM – 10:15 PM*
   3 ☐ At 22:15 *(10:15 PM)* – 00:30 *(12:30 AM)*
   2 ☐ At 00:30 *(12:30 AM)* – 01:45 *(1:45 AM)*
   1 ☐ At 01:45 *(1:45 AM)* – 03:00 *(3 AM)*

3. To get up in the morning at a specific time. How often do you really need your alarm clock to remind you?
   4 ☐ I don't need it
   3 ☐ I don't need it much
   2 ☐ I need it quite a lot
   1 ☐ I need it a lot

4. Do you find it easy to get up in the mornings? (when you're not woken up unexpectedly)
   1 ☐ Not easy at all
   2 ☐ Not very easy
   3 ☐ Quite easy
   4 ☐ Very easy

5. Once you wake up in the morning, how do you feel during the first half hour?
   1 ☐ Nothing to worry about
   2 ☐ Not very alert
   3 ☐ Quite alert
   4 ☐ Very alert

6. Once you wake up in the morning, what is your appetite like during the first half hour?
   1 ☐ Very scarce
   2 ☐ Quite scarce
   3 ☐ Pretty good
   4 ☐ Very good

7. Once you wake up in the morning, how do you feel during the first half hour?
   1 ☐ Very tired
   2 ☐ Quite tired
   3 ☐ Quite rested
   4 ☐ Very rested

8. When you have no commitments the next day, what time do you go to bed compared to your usual bedtime?
   4 ☐ Never or rarely or later
   3 ☐ Less than 1 hour later
   2 ☐ 1 to 2 hours later
   1 ☐ More than 2 hours later

9. You've decided to do some exercise. A friend suggests you do it for an hour twice a week, and according to him, the best time would be between 7 and 8 in the morning. Taking nothing else into account except your own internal clock, how do you think you'd feel?
   4 ☐ I would be in good shape
   3 ☐ It would be in acceptable shape
   2 ☐ I would find it difficult
   1 ☐ I would find it very difficult

10. At what approximate time of night do you feel tired and consequently need to sleep?
   5 ☐ At 8 PM – 9 PM
   4 ☐ At 9 PM – 10:15 PM
   3 ☐ At 22:15 (10:15 PM) – 00:45 (12:45 AM)
   2 ☐ At 00:45 (12:45 AM) – 02:00 (2 AM)
   1 ☐ At 02:00 (2 AM) – 03:00 (3 AM)

11. You want to be at your peak performance for a mentally exhausting two-hour test. You're completely free to plan your day and only think about when you'd feel best. What time would you choose?
    6 ☐ From 08:00 (8 AM) to 10:00 (10 AM)
    4 ☐ From 11:00 (11 AM) to 13:00 (1 PM)
    2 ☐ From 1:00 PM to 5:00 PM
    0 ☐ From 7 PM to 9 PM

12. If you went to bed at 11 pm, what level of tiredness would you notice?
    0 ☐ No tiredness
    2 ☐ Some tiredness
    3 ☐ Quite tired
    5 ☐ Very tired

13. For some reason you went to bed several hours later than usual, even though you don't have to get up at any particular time the next day. When do you think you would wake up?
    4 ☐ At the usual time and I wouldn't sleep anymore
    3 ☐ At the usual time and then I would doze off
    2 ☐ At the usual time and I would go back to sleep
    1 ☐ Later than usual

14. One night you have to stay up from 4 to 6 a.m. due to a night shift. With no commitments the next day, what would you prefer?
    1 ☐ Not going to bed until after the shift
    2 ☐ Take a nap before and sleep after
    3 ☐ Get a good night's sleep before and a nap after
    4 ☐ You would only sleep before the shift

15. You have to do two hours of heavy physical work. You are completely free to plan your day. Thinking only about when you would feel best, what time would you choose?
    4 ☐ From 08:00 (8 AM) to 10:00 (10 AM)
    3 ☐ From 11:00 (11 AM) to 13:00 (1 PM)
    2 ☐ From 1:00 PM to 5:00 PM
    1 ☐ From 7 PM to 9 PM

16. You've decided to do some intense exercise. A friend suggests you exercise for an hour twice a week from 10 to 11 pm. Thinking only about when you would feel best, how do you think it would feel?
    1 ☐ I would be in good shape
    2 ☐ It would be in acceptable shape
    3 ☐ I would find it difficult
    4 ☐ I would find it very difficult

17. Imagine you can choose your work schedule. Suppose your workday is five hours long (including breaks) and that your work is interesting and paid according to your performance. What five consecutive hours would you select? Starting at what time? Consider the rightmost marked box to choose from the following ranges:
    5 ☐ Between 04:00 (4 AM) and 08:00 (8 AM)
    4 ☐ Between 08:00 (8 AM) and 09:00 (9 AM)
    3 ☐ Between 09:00 (9 AM) and 14:00 (2 PM)
    2 ☐ Between 2 PM and 5 PM
    1 ☐ Between 5 PM and 4 AM

18. At what time of day do you think you reach your peak well-being?
    5 ☐ Between 05:00 (5 AM) and 08:00 (8 AM)
    4 ☐ Between 08:00 (8 AM) and 10:00 (10 AM)
    3 ☐ Between 10:00 (10 AM) and 17:00 (5 PM)
    2 ☐ Between 5 PM and 10 PM
    1 ☐ Between 10 PM and 5 AM

19. People are often described as morning types and evening types. Which of these types do you consider yourself to be?
    6 ☐ A clearly morning type.
    4 ☐ More of a morning person than an evening person.
    2 ☐ A more evening type than an early type.
    0 ☐ A clearly evening type.

Add up the points shown next to the box.
The score obtained was: ________ points.
