# Supplementary material for: Association Between Chronotype and Cardiometabolic Risk in 1462 Adults from the General Population: Mediation Analysis of Body Fat Percentage and Waist-to-Height Ratio
Source: Metabolites. 2026 Apr 4;16(4):243. doi: 10.3390/metabo16040243 (PMC13118073; doi:10.3390/metabo16040243)
Supplement: Supplementary file 1 [file metabolites-16-00243-s001.zip › Supplementary Material S2. Ethical Approval Letter.pdf]

UNAP
Universidad Nacional de la Amazonía Peruana

COMITÉ INSTITUCIONAL DE ÉTICA EN INVESTIGACIÓN-(CIEI)

"Año del Bicentenario, de la consolidación de nuestra Independencia, y de la conmemoración de las heroicas batallas de Junín y Ayacucho"

DICTAMEN DE EVALUACIÓN Nº 018-2024-CIEI-VRINV-UNAP

Iquitos, 27 de febrero de 2024

Ingº JORGE ARMANDO SIFUENTES DA SILVA
Investigador Principal – Facultad de Industrias Alimentarias
Maestría en Gestión Alimentaria con mención en Nutrición
Escuela de Postgrado de la UNAP

TÍTULO DEL PLAN DE TESIS: ***"CRONOTIPO, GRASA CORPORAL, ESTADO NUTRICIONAL ANTROPOMÉTRICO Y RIESGO CARDIOMETABÓLICO EN ADULTOS DE LA CIUDAD DE IQUITOS, 2024";*** recepcionado el 19 de febrero de 2024.

UNIVERSIDAD NACIONAL DE LA AMAZONIA PERUANA
COMITÉ DE ÉTICA
CIEI-UNAP
IQUITOS

Código asignado por el Comité:

Le informo que el proyecto de referencia ha sido evaluado por el Comité obteniendo los resultados que se describen a continuación:

	**Nº Y FECHA VERSIÓN**	**DECISIÓN**
PROTOCOLO	PI-018-27/02/24-CIEI-UNAP	(1)
CONSENTIMIENTO INFORMADO	CI-018-27/02/24-CIEI-UNAP	(1)

Se concluye que:
Ha sido **APROBADO SIN MODIFICACIONES EN EL PROTOCOLO (1) Y EN EL CONSENTIMIENTO INFORMADO (1).**

Este protocolo tiene vigencia del 27/02/2024 hasta 27/08/2024, por un periodo de 6 meses.

En caso de requerir una ampliación, le rogamos tenga en cuenta que deberá enviar al Comité Institucional de Ética en Investigación de la Universidad Nacional de la Amazonía Peruana (CIEI-UNAP), un reporte de progreso al menos 30 días antes de la fecha de término de su vigencia.

El Comité dispone de un formato estándar que podrá usarse al efecto, ubícanos al correo electrónico: comite_etica@unapiquitos.edu.pe.

OBSERVACIONES AL PROTOCOLO

1. El Plan de Investigación, titulado: ***"CRONOTIPO, GRASA CORPORAL, ESTADO NUTRICIONAL ANTROPOMÉTRICO Y RIESGO CARDIOMETABÓLICO EN ADULTOS DE LA CIUDAD DE IQUITOS, 2024";*** fue **Aprobado sin Modificación en el Protocolo con valoración (1)**, sin ninguna observación.

Escaneado con CamScanner

OBSERVACIONES AL CONSENTIMIENTO INFORMADO

1. El Plan de Investigación, titulado: ***"CRONOTIPO, GRASA CORPORAL, ESTADO NUTRICIONAL ANTROPOMÉTRICO Y RIESGO CARDIOMETABÓLICO EN ADULTOS DE LA CIUDAD DE IQUITOS, 2024";*** fue **Aprobado sin Modificación en el Consentimiento Informado con valoración (1),** sin ninguna observación, respecta la privacidad y confidencialidad de los sujetos de investigación.

CONCLUSIÓN

- Los Miembros del CIEI-UNAP manifiestan no tener conflictos de interés con la evaluación de este estudio.
- Procede la **ejecución de la investigación.**

Atentamente,

UNIVERSIDAD NACIONAL DE LA AMAZONIA PERUANA
COMITÉ DE ETICA CIEI-UNAP
IQUITOS

HERMANN FEDERICO SILVA DELGADO
Presidente
Comité Institucional de Ética en Investigación – UNAP

Nota:
- La Tasa por Servicio de Evaluación del CIEI-UNAP, se realizó por cuatrocientos uno con 00/100 soles (S/. 401.00) con el Voucher Nº 0687538, efectuado en el Banco de la Nación.

C.c.: Interesado, Archivo.
Uliveth

Escaneado con CamScanner
