# Supplementary material for: Association Between Chronotype and Cardiometabolic Risk in 1462 Adults from the General Population: Mediation Analysis of Body Fat Percentage and Waist-to-Height Ratio
Source: Metabolites. 2026 Apr 4;16(4):243. doi: 10.3390/metabo16040243 (PMC13118073; doi:10.3390/metabo16040243)
Supplement: Supplementary file 1 [file metabolites-16-00243-s001.zip › Supplementary Material S3. Written informed consent.pdf]

CONSENTIMIENTO INFORMADO

Presentación

Señor(a), estoy realizando un estudio titulado: "Cronotipo, grasa corporal, estado nutricional y riesgo cardiometabólico de adultos de la ciudad de Iquitos, 2024", con el objetivo de determinar el tipo de cronotipo que tiene una persona, se medirá el porcentaje de grasa corporal, como el estado nutricional que este presenta, y buscar la relación que estas variables con el riesgo cardiometabólico.

Los datos obtenidos, serán analizados de forma anónima, y agrupada, no se manejará datos individuales. Posterior al procesamiento de los datos, y la sustentación del trabajo de investigación, estos serán destruidos, protegiendo la integridad física y moral de cada participante del estudio. Los resultados de este proyecto serán de conocimiento solo de del equipo de investigación y los resultados finales estarán a disposición mediante publicaciones y sus datos personales no serán revelados a terceros en ningún momento, su participación en este estudio es de suma importancia porque permitirá tener datos reales con respecto a las variables de estudios, asimismo, estos resultados, ayudaran a las autoridades pertinentes a buscar estrategias para promocionar el buen cuidado de la salud.

Yo, ____he leído y comprendido la información anterior y mis preguntas han sido respondidas de manera satisfactoria. He sido informado y entiendo que los datos obtenidos en el estudio pueden ser publicados o difundidos con fines científicos. Convengo en aceptar mi participación en el presente estudio.

Recibiré una copia firmada y fechada de esta forma de consentimiento.

____

Firma del participante

Código de Identificación:

DNI:

____

Firma del investigador

Nombre y apellidos:

DNI:

____

FECHA
