# Supplementary material for: Association Between Chronotype and Cardiometabolic Risk in 1462 Adults from the General Population: Mediation Analysis of Body Fat Percentage and Waist-to-Height Ratio
Source: Metabolites. 2026 Apr 4;16(4):243. doi: 10.3390/metabo16040243 (PMC13118073; doi:10.3390/metabo16040243)
Supplement: Supplementary file 1 [file metabolites-16-00243-s001.zip › Supplementary Material S4. Collinearity diagnostics for predictors included in Model 3.pdf]

Supplementary Table S2. Collinearity diagnostics for predictors included in Model 3

**Variable**	**Tolerance**	**VIF**
Chronotype score	0.981	1.020
Age	0.982	1.018
Sex	0.912	1.096
Smoking status	0.862	1.160
Family history of cardiometabolic disease	0.849	1.177
Physical activity level	0.848	1.179
Body fat percentage	0.717	1.395
Waist-to-height ratio	0.680	1.471

Note: Multicollinearity was assessed using tolerance statistics and variance inflation factors (VIF). No problematic multicollinearity was detected (VIF range: 1.02–1.47; tolerance range: 0.68–0.98).
