# Supplementary material for: Association Between Chronotype and Cardiometabolic Risk in 1462 Adults from the General Population: Mediation Analysis of Body Fat Percentage and Waist-to-Height Ratio
Source: Metabolites. 2026 Apr 4;16(4):243. doi: 10.3390/metabo16040243 (PMC13118073; doi:10.3390/metabo16040243)
Supplement: Supplementary file 1 [file metabolites-16-00243-s001.zip › Supplementary Material S5. Pairwise correlations among predictors.pdf]

Supplementary Table S1. Pairwise correlations among predictors.

Variable	Chronotype	Age	Sex	Smoking	FH-CMD	PAL	Body fat %	WHtR
**Chronotype**	1	0.063*	0.077*	-0.043	0.014	-0.022	-0.064*	-0.004
**Age**	0.063*	1	-0.044	-0.039	-0.061*	-0.046	0.080*	0.030
**Sex**	0.077*	-0.044	1	0.057*	0.035	-0.050	-0.139*	-0.273*
**Smoking**	-0.043	-0.039	0.057*	1	0.296*	0.290*	0.001	-0.045
**FH-CMD**	0.014	-0.061*	0.035	0.296*	1	0.317*	-0.019	-0.045
**PAL**	-0.022	-0.046	-0.050	0.290*	0.317*	1	-0.001	0.038
**Body fat %**	-0.064*	0.080*	-0.139*	0.001	-0.019	-0.001	1	0.513*
**WHtR**	-0.004	0.030	-0.273*	-0.045	-0.045	0.038	0.513*	1

*$p < 0.05$. FH-CMD = Family history of cardiometabolic disease; PAL = Physical activity level; WHtR = Waist-to-height ratio.
